# Supplementary material for: A default prior for regression coefficients
Source: Stat Methods Med Res. 2018 Dec 13;28(12):3799–807. doi: 10.1177/0962280218817792 (PMC6745606; doi:10.1177/0962280218817792)
Supplement: Supplemental Material1 - Supplemental material for A default prior for regression coefficients [file Supplemental_Material1.pdf]

Supplement to: A default prior for regression coefficients

Erik van Zwet

September 21, 2018

Contents

Preliminaries **1**

Section 1 Introduction; Figure 1 **1**

Section 2.2 Parameterization invariance; Figure 2 **2**

Section 3.1 MEDLINE; Figure 3 **3**

Section 3.2 Results **4**
Marginal model 5

Session information **6**

Preliminaries

Set some display options

```
options(width = 90)
```

Section 1 Introduction; Figure 1

```
b=seq(0,3.29,0.01)  # tau = sigma, then theta|x ~ N(x/2,1/sqrt(2))
p=2*pnorm(-abs(b))
coverage=pnorm(b+1.96,b/2,1/sqrt(2)) - pnorm(b-1.96,b/2,1/sqrt(2))
plot(p,coverage,type='l',bty="l",ylab='Conditional coverage',xlab='p-value',log="x")
axis(side=1,at=1)
abline(h=0.95)
```

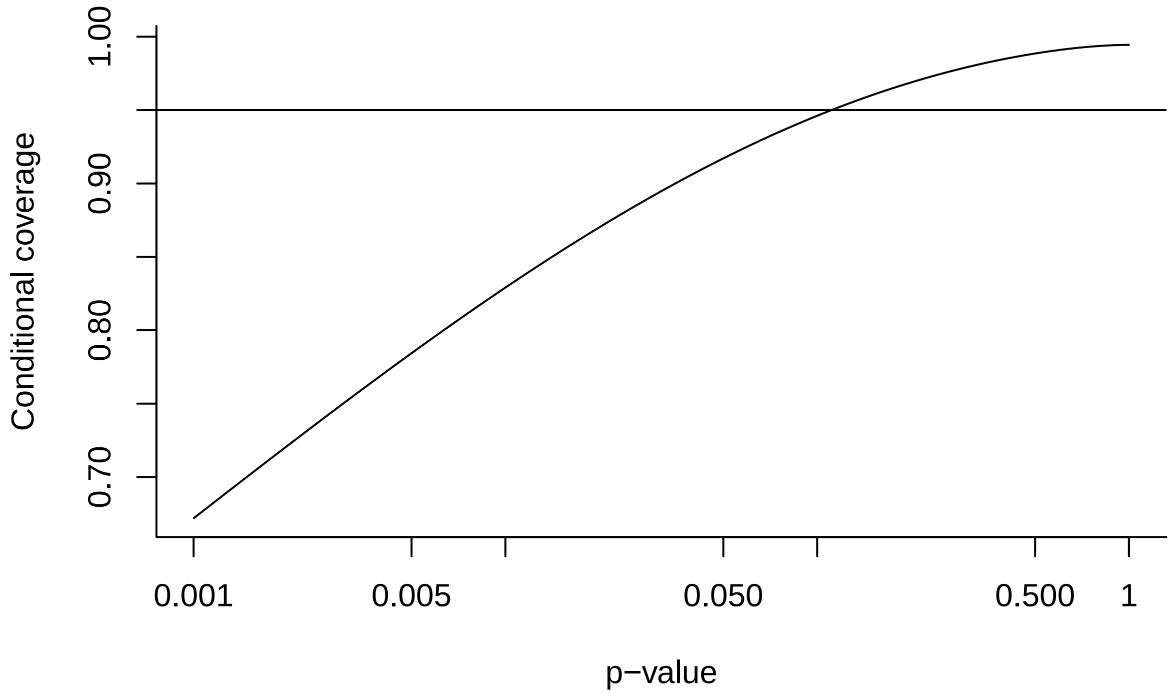

Section 2.2 Parameterization invariance; Figure 2

```
set.seed=123
theta=seq(0,5,0.1)
k=length(theta)
n=100000
plot(0,0,bty='l',type='n',xlim=c(0,5),ylim=c(0,1),xlab="theta",ylab="Jeffreys")
for (sigma in c(0.5,1,2)){
  dll=matrix(NA,n,k)
  for (i in 1:n){
    x=theta+rnorm(1,0,sigma)
    lik=dnorm(x,theta,sigma) + dnorm(x,-theta,sigma)  # likelihood
    dll[i,]=((x-theta)*dnorm(x,theta,sigma)/sigma     # derivative of the log lik.
             - (x+theta)*dnorm(x,-theta,sigma)/sigma)/lik
  }
  Jeffreys=sqrt(apply(dll^2,2,mean))
  lines(theta,Jeffreys,bty='l',type='l', xlim=c(0,5),ylim=c(0,1))
}
```

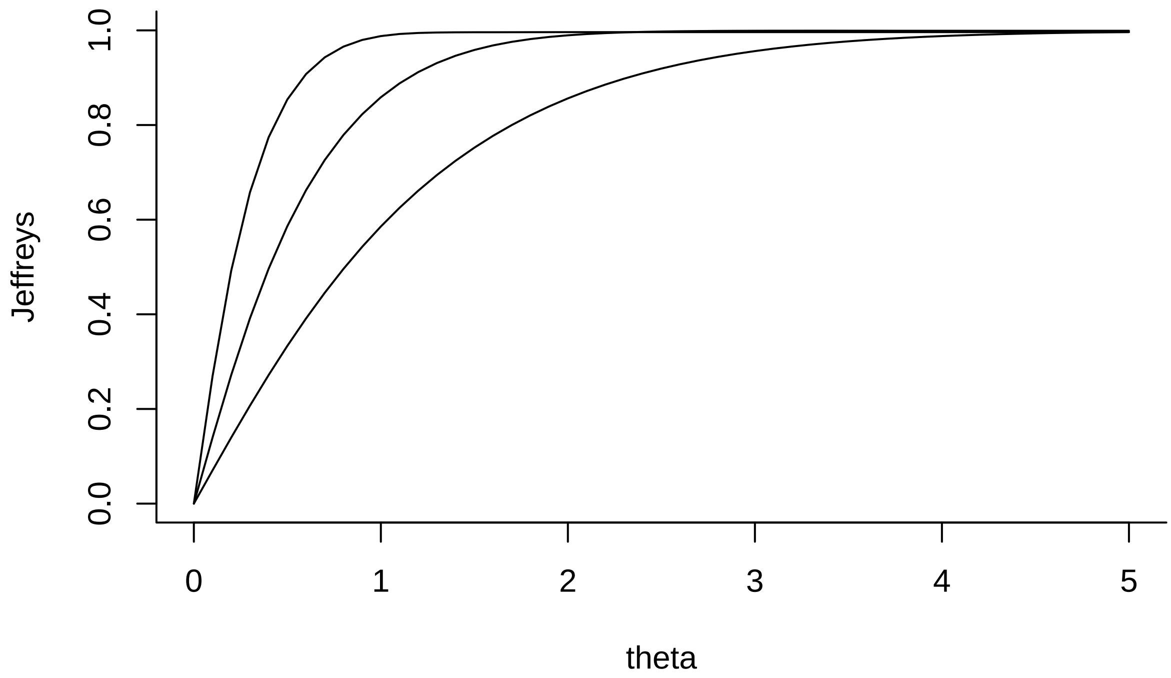

Section 3.1 MEDLINE; Figure 3

```
#d=read.csv("M:\\papers\\regression coefficient\\SMMR\\supplement\\MEDLINE.csv",header=TRUE)
d=read.csv("MEDLINE.csv",header=TRUE)
length(unique(d$PMID))    # 80 studies in total
```

```
## [1] 80
```

```
d=d[!is.na(d$pvalue),]
d$PMID=factor(d$PMID)
length(unique(d$PMID))    # 50 studies with data
```

```
## [1] 50
```

```
d$z=abs(qnorm(d$pvalue/2))
hist(d$z,main="",xlab='absolute Z-value')
```

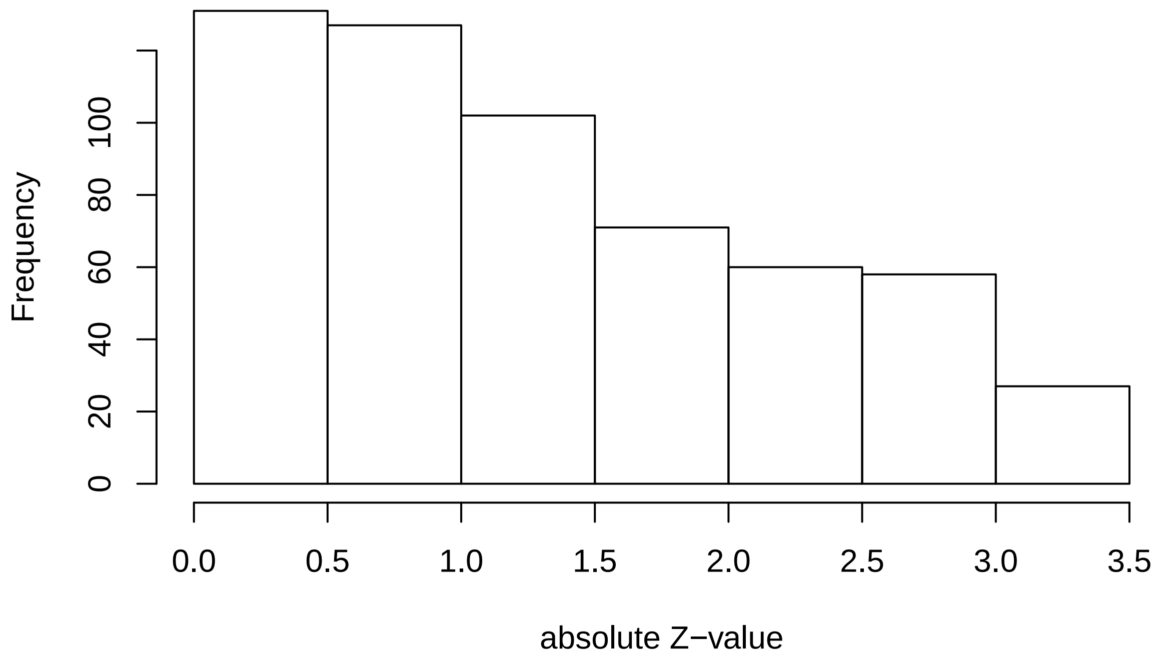

Section 3.2 Results

Recall that if Z has the normal distribution with men zero and variance σ^2, then Z^2 has the gamma distribution with shape $1/2$ and scale $2\sigma^2$. For regression modeling, this distribution is reparameterized in terms of its shape and its mean σ^2 (the product of shape and scale). Also note that the variance of this distribution is equal to two times the square of the mean.

```
library(glmmTMB)
d$offset=1                # offset for mean
d$disp=log(1/2)           # offset for dispersion (set shape to 1/2)
fit=glmmTMB(z^2 ~ 1 + offset(offset) + (1 | PMID), dispformula=~0+offset(disp),
            data=d, family=Gamma("identity"))
summary(fit)
```

```
##  Family: Gamma  ( identity )
## Formula:          z^2 ~ 1 + offset(offset) + (1 | PMID)
## Dispersion:           ~0 + offset(disp)
## Data: d
##
##      AIC      BIC   logLik deviance df.resid
##   2013.1   2021.8  -1004.5   2009.1      574
##
## Random effects:
##
## Conditional model:
```

```
##  Groups Name        Variance Std.Dev.
##  PMID   (Intercept) 0.4675   0.6838
## Number of obs: 576, groups:  PMID, 50
##
## Conditional model:
##             Estimate Std. Error z value Pr(>|z|)
## (Intercept)    1.638      0.199    8.23   <2e-16 ***
## ---
## Signif. codes:  0 '***' 0.001 '**' 0.01 '*' 0.05 '.' 0.1 ' ' 1
```

```
CI=confint(fit)
CI
```

```
##                                   2.5 %   97.5 %  Estimate
## cond.(Intercept)              1.2479963 2.028247 1.6381218
## cond.Std.Dev.PMID.(Intercept) 0.4299157 1.087472 0.6837552
```

```
sqrt(CI[1,])
```

```
##    2.5 %   97.5 % Estimate
## 1.117138 1.424165 1.279891
```

Marginal model

As an alternative to the (conditional) mixed model, we can fit a marginal model. We do not report this model in the paper, but include it here to show that the results are very similar.

Note that by setting “scale.value=2”, we are specifying that the variance is equal to 2 times the square of the mean.

```
library(geepack)
fit=geese(z^2 ~ 1 + offset(offset), id=PMID, scale.fix=TRUE, scale.value=2,
          corstr="exchangeable",data=d, family=Gamma("identity"))
summary(fit)
```

```
##
## Call:
## geese(formula = z^2 ~ 1 + offset(offset), id = PMID, data = d,
##     family = Gamma("identity"), scale.fix = TRUE, scale.value = 2,
##     corstr = "exchangeable")
##
## Mean Model:
##  Mean Link:                 identity
##  Variance to Mean Relation: Gamma
##
##  Coefficients:
##             estimate    san.se     wald p
## (Intercept)  1.61414 0.1871599 74.38011 0
##
## Scale is fixed.
##
## Correlation Model:
##  Correlation Structure:     exchangeable
##  Correlation Link:          identity
##
##  Estimated Correlation Parameters:
```

```
##         estimate     san.se     wald           p
## alpha 0.02841132 0.01073489 7.004665 0.008129756
##
## Returned Error Value:    0
## Number of clusters:   50   Maximum cluster size: 37
```

Session information

```
sessionInfo()
```

```
## R version 3.5.1 (2018-07-02)
## Platform: x86_64-w64-mingw32/x64 (64-bit)
## Running under: Windows 10 x64 (build 17134)
##
## Matrix products: default
##
## locale:
## [1] LC_COLLATE=Dutch_Netherlands.1252  LC_CTYPE=Dutch_Netherlands.1252
## [3] LC_MONETARY=Dutch_Netherlands.1252 LC_NUMERIC=C
## [5] LC_TIME=Dutch_Netherlands.1252
##
## attached base packages:
## [1] stats     graphics  grDevices utils     datasets  methods   base
##
## other attached packages:
## [1] geepack_1.2-1   glmmTMB_0.2.2.0
##
## loaded via a namespace (and not attached):
##  [1] Rcpp_0.12.18    lattice_0.20-35 digest_0.6.16   rprojroot_1.3-2 MASS_7.3-50
##  [6] grid_3.5.1      nlme_3.1-137    backports_1.1.2 magrittr_1.5    evaluate_0.11
## [11] stringi_1.1.7   minqa_1.2.4     nloptr_1.0.4    Matrix_1.2-14   rmarkdown_1.10
## [16] splines_3.5.1   lme4_1.1-18-1   tools_3.5.1     TMB_1.7.14      stringr_1.3.1
## [21] yaml_2.2.0      compiler_3.5.1  htmltools_0.3.6 knitr_1.20
```
